# Current globalization of drug interventional clinical trials: characteristics and associated factors, 2011–2013

**DOI:** 10.1186/s13063-017-2025-1

**Published:** 2017-06-21

**Authors:** Sohyun Jeong, Minji Sohn, Jae Hyun Kim, Minoh Ko, Hee-won Seo, Yun-Kyoung Song, Boyoon Choi, Nayoung Han, Han-Sung Na, Jong Gu Lee, In-Wha Kim, Jung Mi Oh, Euni Lee

**Affiliations:** 1 College of Pharmacy and Research Institute of Pharmaceutical Sciences, Seoul National University, Seoul, Korea; 2 College of Pharmacy, Sungkyunkwan University, Suwon, Korea; 3 Clinical Research Division, Toxicology Evaluation and Research Department, National Institute of Food and Drug Evaluation, Chungcheongbuk-do, Korea

**Keywords:** Clinical trials, Intellectual property rights index, Economic freedom index, Human capital index, Health care expenditure per capita

## Abstract

**Background:**

Clinical trial globalization is a major trend for industry-sponsored clinical trials. There has been a shift in clinical trial sites towards emerging regions of Eastern Europe, Latin America, Asia, the Middle East, and Africa. Our study objectives were to evaluate the current characteristics of clinical trials and to find out the associated multiple factors which could explain clinical trial globalization and its implications for clinical trial globalization in 2011–2013.

**Methods:**

The data elements of “phase,” “recruitment status,” “type of sponsor,” “age groups,” and “design of trial” from 30 countries were extracted from the ClinicalTrials.gov website. Ten continental representative countries including the USA were selected and the design elements were compared to those of the USA. Factors associated with trial site distribution were chosen for a multilinear regression analysis.

**Results:**

The USA, Germany, France, Canada, and United Kingdom were the “top five” countries which frequently held clinical trials. The design elements from nine continental representative countries were quite different from those of the USA; phase 1 trials were more prevalent in India (OR 1.517, *p* < 0.001) while phase 3 trials were much more prevalent in all nine representative countries than in the USA. A larger number of “child” age group trials was performed in Poland (OR 1.852, *p* < 0.001), Israel (OR 1.546, *p* = 0.005), and South Africa (OR 1.963, *p* < 0.001) than in the USA. Multivariate analysis showed that health care expenditure per capita, Economic Freedom Index, Human Capital Index, and Intellectual Property Rights Index could explain the variance of regional distribution of clinical trials by 63.6%.

**Conclusions:**

The globalization of clinical trials in the emerging regions of Asia, South Africa, and Eastern Europe developed in parallel with the factors of economic drive, population for recruitment, and regulatory constraints.

**Electronic supplementary material:**

The online version of this article (doi:10.1186/s13063-017-2025-1) contains supplementary material, which is available to authorized users.

## Background

Clinical trials are fundamental platforms for the development of clinical guidelines and improved clinical practice in health care systems. Traditionally, clinical trials have been carried out in wealthy countries of North America, Western Europe, and the Oceania regions. However, there has been a shift in clinical trial sites towards emerging regions of Eastern Europe, Latin America, Asia, the Middle East, and Africa, with benefits including lower costs and faster patient recruitment to the pharmaceutical companies. This shift between 1990 and 2000 was evident especially when the US Food and Drug Administration (FDA) started focusing on tracking clinical drug trials outside the USA [1–3]. For example, it was reported that pharmaceutical companies were able to complete phase 3 trials up to 6 to 7 months earlier in low-cost countries [4, 5]. As an early market entry is crucial to guaranteeing huge advantages to a new drug within its particular therapeutic area [6], finding a clinical trial site with fast patient recruitment has become a highly promising aspect of industrial sponsorship for speedy drug development.

In 2007, over 60% of pivotal studies submitted to the Center for Drug Evaluation and Research of the US FDA contained data gathered from one or more foreign study sites. As a result, clinical trials for medical products were completed at nearly 6500 foreign sites from October 2007 to September 2008 [7]. Previous studies [8, 9] focused mainly on the economic issues. However, the factors contributing to globalization and wide geographical distribution of clinical trials include patient pool, regulatory conditions, relevant expertise, infrastructure, and environment, in addition to substantial cost savings [4, 10]. Thus, a multifactorial assessment is required in order to identify and understand the current characteristics and trends of the wide geographical distribution of clinical trials.

As the most accessible and largest registry of clinical trials worldwide, the first version of ClinicalTrials.gov was made publicly available from year 2000 and the number of registered clinical trials in ClinicalTrials.gov has increased to 190,253 studies [11]. The trial registry presents its database as recommended by World Health Organization (WHO) [12] in the format of the WHO-mandated Trial Registration Data Set (TRDS) which can be easily used for analysis [13]. Califf et al. [11] reported the characteristics of clinical trials registered in ClinicalTrials.gov between 2007 and 2010 which focused on factors associated with the use of randomization, blinding, and data monitoring committees. Our study aimed to evaluate the different aspects of the characteristics of drug intervention clinical trials, thereby exploring the implications of the globalization of clinical trials in 2011–2013.

## Methods

### Clinical trial data collection

The information of each clinical trial, as registered by geographical location of countries and regions, was obtained and downloaded from the ClinicalTrials.gov website [14]. The “top 30” countries performing the largest number of clinical trials, with continental representation from North America, South America, Western Europe, Eastern Europe, the Middle East, East Asia, South Asia, Pacifica, and Africa, were selected based on the data available from the section of “trends, charts, and maps.” For comparative evaluations by the data elements of clinical trials, 10 countries were selected. Eight countries that conducted the largest number of clinical trials in their respective continental region were selected as representatives of their respective continents; the USA in North America, Brazil in South America, Germany in Western Europe, Poland in Eastern Europe, Israel in the Middle East, Korea in Asia, Australia in Pacifica, and South Africa in Africa. An additional two countries, China and India, from Asia were included in the analysis due to the fact that they are the two most cited countries in the academic literature [15–17] concerned with globalization of clinical trials. Design elements of clinical trials of nine representative countries were compared with those of the USA. The largest number of clinical trials are conducted in the USA and five out of the “top 10” pharmaceutical industries are in the USA [18]; therefore, the characteristics of clinical trials of the USA were set as the comparison target.

### Data extraction

The total number of trials during 2011–2013 from the top 30 countries and the information about the trials including the name of the trial, identification number, recruitment status, registration date, phase of the trial, sponsorship, and other characteristics were collected. Among the 20 items of the TRDS, the data were extracted and categorized as “phase of the trials (from phase 0 to phase 4),” “recruitment status (completed, recruiting, terminated, withdrawn, suspended, active not recruiting, enrolling by invitation),” “type of sponsor (industry, NIH or US federal agency, others),” “age of participant (child, adult, senior, adult to senior, child to senior),” and “design of randomization.” When the sponsorship designation included multiple entities, the first indicated sponsorship category was considered as the primary sponsor in our study.

The factors (explanatory variables) associated with globalization of clinical trials were collected as representing indices of the host country, economic index: gross domestic product (GDP) [18] and health care expenditure per capita (HEC) in US dollars [19], market capacity: population in million (PIM) [20] and HEC, infrastructure: rank of Human Capital Index (HCI) [21], Economic Freedom Index (EFI) [22], Intellectual Property Rights Index (IPRI) [23], and bureaucracy: EFI and IPRI. The higher score represents better performance for each explanatory variable except for HCI and IPRI (Additional file 1).

### Outcomes

The primary outcome of our study was to describe the characteristics of clinical trials in the top 30 countries by the registered data elements of study phase, recruitment status, age groups, and type of sponsorship and design of trials in 2011–2013. The secondary outcome was to evaluate the associations between the number of clinical trials in geographical distribution and explanatory variables of GDP, HEC, PIM, HCI, EFI, and IPRI. In addition, data elements of clinical trial between the USA and nine continental representative countries were assessed.

### Statistical analyses

Multiple linear regression analysis with backward elimination was employed to build prediction models for the factors affecting the geographical distribution of clinical trials, using a *p* value of < 0.1. For all models, partial regression coefficient estimates (*β*), 95% confidence interval (CI), significance test results (*p* values), and percentage variance accounted for by the model as a whole (*R* ^2^) were reported. Residuals from the final models were examined to ensure that their distributions reasonably satisfied model assumptions of normality and homoscedasticity. For the comparison between the USA and nine continental representative countries, the odds ratios (ORs), and *p* values were presented by chi-square analysis. Analyses were performed using SPSS Version 21.0 (SPSS, Inc., Chicago, IL, USA). All statistical tests were two-tailed, and *p* < 0.05 was considered as statistically significant.

## Results

### Trends and characteristics of clinical trials in the top 30 countries

**Fig. 1:**
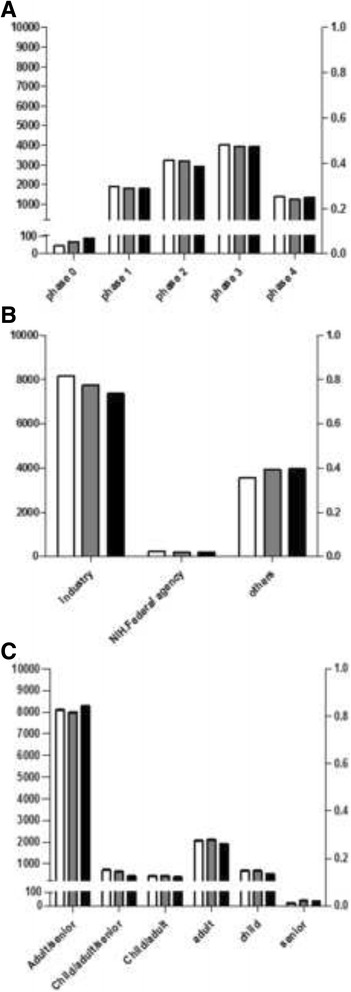
The change of drug intervention clinical trials by phase, sponsor type and age groups in 2011–2013 (**a**) phases, (**b**) sponsor types, (**c**) age groups. Y *left* axis indicates the number of clinical trials and Y *right* axis indicates the proportion of clinical trials. Each pattern designates the registration year of clinical trials; 2011, 2012, 2013

The total number of registered drug intervention clinical trials was 35,393. The frequency and features of drug interventional clinical trials in 30 countries are shown in Additional file 2. The overall change in the total number and in each data element of clinical trials from 2011 to 2013 was not remarkable. The total number was slightly decreased to 11,537 from 11,986. However, the number of phase 0 trials showed consistent increase from 2011 to 2013. Industrial sponsorships slightly decreased, whereas sponsorships of others showed an increase. Clinical trials for the “child” age group were decreased to 28.25% in 2013 compared to 35.93% in 2011 (Fig. 1).

The “top five” countries that most frequently held the trials were the USA (10,473), Germany (2079), France (1863), Canada (1859), and the United Kingdom (1787), which were located in traditional regions of North America and Western Europe, altogether hosting 51% of all clinical trials (18,061 (sum of five countries)/35,393 (total number of clinical trials)). A large portion of clinical trials were held in the USA (29.59%) followed by Germany (5.87%). Except for the USA, each country contributed less than 6% of the total number of clinical trials during the study period. Among countries in Asia, Korea (6th) and China (8th) made the ranks within the top 10 countries. Each country that ranked beyond 20th held fewer than 500 clinical trials during the 3-year study period and their sum of clinical trials represented 8.3% (2943/35,393) out of total clinical trials (Additional file 2).

The sum of phase 2 and 3 trials occupied over 60% (21,241/35,393) of total clinical trials, while phase 4 trials took the proportion of 11.16% (3,951/35,393). Most of the phase 0 trials were held in the USA, representing 78.98% (145/196) out of all phase 0 trials. Completed trials were 36.30% (11,027/35,393) and the proportion of clinical trials that were withdrawn, terminated, or suspended was 5.53% (1959/35,393). The industrial sponsorship was the most dominant type of the sponsorship, representing 65.80% (23,285/35,393). NIH or other US Federal Agency sponsored clinical trials were only 1.81% (639/35,393), while investigator and individual organization sponsorships that were designated as “others” occupied 32.40% (11,469/35,393). The age group of adult/senior was the highest subject age group, 68.8% (24,351/35,393), while child group and senior group showed only 5.52% (1848/35,393) and 0.002% (86/35,393), respectively (Additional file 2).

### Regression analyses of clinical trial site distribution with explanatory variables

Multiple linear regression analyses between the number of clinical trials and explanatory variables (PIM, GDP, HEC, EFI, HCI, and IPRI) were performed to identify significant factors affecting the geographical distribution of number of clinical trials (Additional file 3). Using the log transformed data to attain normal distribution, variables were entered in the multiple regression analysis with enter, stepwise, forward inclusion, and backward elimination methods consecutively. From the analyses, backward elimination method generated the highest correlation coefficient (adjusted *R* ^2^ 0.636, *p* < 0.001), inferring that this model could explain 63.6% of the variance in the number of clinical trials observed in the top 30 countries. Among the variables, GDP and PIM showed the multicollinearity by the backward elimination method, and the final regression model included IPRI, EFI, HCI, and HEC (Table 1).

**Table 1:** The explanatory variables in relation to geographical distribution of the number of clinical trials

Final model: *F* = 12.777, *p* = 0.000; *R* ^2^ = 0.690 (adjusted *R* ^2^ = 0.636)					
Variables^a^	Estimate (*β*)	95% confidence interval for *β*	Standardized *β*	Standard error	*p* value
Constant	−8.536	−19.248, 2.176		5.178	0.113
EFI	3.210	0.637, 5.783	0.485	1.244	0.017
HEC	0.341	0.049, 0.633	0.415	0.141	0.024
HCI	0.618	0.304, 0.932	0.759	0.152	0.000
IPRI	−0.746	−0.997, 0.496	0.730	0.121	0.000

^a^Variables: *EFI* Economic Freedom Index, *HEC* Health Care Expenditure per Capita, *HCI* Human Capital Index, *IPRI* Intellectual Property Rights Index

### Comparative characteristics of clinical trials between the USA and nine continental representative countries

**Fig. 2:**
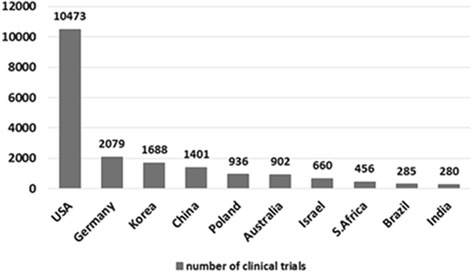
The number of clinical trials from 10 countries selected in the comparative analysis

For in-depth evaluations of the clinical trials by design elements, top 10 countries were selected. Their contributions and key characteristics on drug research and development were further evaluated. The number of clinical trials from each representative country was tabulated in descending order and its continent-specific contribution is portrayed in a graphical display (Fig. 2).

The design elements for clinical trials were compared using the USA as a reference (Table 2).

**Table 2:** Comparison of design elements of drug or biological interventional trials between 13 regional representative countries and USA in 2011 − 2013 (odds ratio, 95% CI, *p* value)

Continental region	South America	Western Europe	Eastern Europe	Middle East	Asia			Pacifica	Africa
Country	Brazil	Germany	Poland	Israel	Korea	China	India	Australia	South Africa
Phase									
0	0.253 (0.035–1.818) *p* = 0.219	-	-	0.547 (0.224–1.339) *p* = 0.180	**0.128** **(0.041–0.403)** ***p*** **< 0.001**	0.567 (0.306–1.049) *p* = 0.067	-	**0.080** **(0.011–0.573)** ***p*** **= 0.001**	**-**
1	**0.330** **(0.215–0.505)** ***p < 0.001***	**0.729** **(0.647–0.820)** ***p < 0.001***	**0.127** **(0.087–0.184)** ***p < 0.001***	**0.223** **(0.159–0.312)** ***p < 0.001***	**0.656** **(0.573–0.751)** ***p < 0.001***	**0.379** **(0.315–0.456)** ***p < 0.001***	**1.517** **(1.206–1.907)** ***p < 0.001***	**0.566** **(0.467–-0.686)** ***p < 0.001***	**0.287** **(0.200–0.411)** ***p < 0.001***
2	**0.402** **(0.280–0.577)** ***p < 0.001***	1.022 (0.925–1.128) *p < 0.673*	1.031 (0.896–1.185) *p = 0.672*	**0.684** **(0.564–0.829)** ***p < 0.001***	**0.715** **(0.633–0.808)** ***p < 0.001***	**0.815** **(0.718–0.925)** ***p = 0.002***	**0.471** **(0.335–0.662)** ***p < 0.001***	0.889 (0.765–-1.033) *p = 0.123*	0.807 (0.650–1.00) *p = 0.050*
3	**1.951** **(1.526–2.494)** ***p < 0.001***	**2.336** **(2.118–2.577)** ***p < 0.001***	**3.557** **(3.176–4.029)** ***p < 0.001***	**2.798** **(2.414–3.243)** ***p < 0.001***	**1.961** **(1.751–2.192)** ***p < 0.001***	**1.596** **(1.404–1.815)** ***p < 0.001***	1.118 (0.822–1.521) *p = 0.477*	**2.968** **(2.614–-3.370)** ***p < 0.001***	**3.349** **(2.840–3.950)** ***p < 0.001***
4	**2.868** **(2.233–3.683)** ***p < 0.001***	**0.797** **(0.675–0.942)** ***p = 0.008***	0.822 (0.649–1.041) *p = 0.104*	1.064 (0.829–1.365) *p = 0.628*	**1.783** **(1.557–2.043)** ***p < 0.001***	**2.142** **(1.865–2.458)** ***p < 0.001***	1.099 (0.758–1.593) *p = 0.618*	**0.725** **(0.562–-0.935)** ***p = 0.013***	1.033 (0.765–1.397) *p = 0.831*
Sponsors									
Industry	**0.592** **(0.461–0.759)** ***p < 0.001***	**4.252** **(3.800–4.760)** ***p < 0.001***	**1.976** **(1.792–2.179)** ***p < 0.001***	**1.385** **(1.222–1.570)** ***p < 0.001***	**1.241** **(1.140–1.352)** ***p < 0.001***	**0.795** **(0.716–0.884)** ***p < 0.001***	**1.257** **(1.036–1.526)** ***p = 0.020***	**1.813** **(1.638–2.006)** ***p < 0.001***	**1.827** **(1.591–2.098)** ***p < 0.001***
Others	**1.534** **(1.278–1.842)** ***p < 0.001***	**0.289** **(0.258–0.324)** ***p < 0.001***	**0.110** **(0.082–0.147)** ***p < 0.001***	**0.714** **(0.611–0.835)** ***p < 0.001***	**0.863** **(0.785–0.950)** ***p = 0.002***	**1.322** **(1.206–1.448)** ***p < 0.001***	0.842 (0.674–1.052) *p = 0.130*	**0.259** **(0.212–-0.317)** ***p < 0.001***	**0.230** **(0.171–0.310)** ***p < 0.001***
Age									
Adult	**1.679** **(1.335–2.111)** ***p < 0.001***	**0.821** **(0.729–0.925)** ***p = 0.001***	**0.379** **(0.299–0.480)** ***p < 0.001***	**0.586** **(0.465–0.738)** ***p < 0.001***	0.968 (0.856–1.094) *p = 0.617*	1.015 (0.890–1.157) *p = 0.825*	**2.695** **(2.211–3.285)** ***p < 0.001***	**0.556** **(0.454–0.682)** ***p < 0.001***	**0.585** **(0.444–0.772)** ***p < 0.001***
Child	0.950 (0.541–1.668) *p = 0.857*	1.001 (0.804–1.248) *p = 0.989*	**1.852** **(1.448–2.369)** ***p < 0.001***	**1.546** **(1.141–2.094)** **p = 0.005**	0.987 (0.775–1.256) *p = 0.914*	1.011 (0.780–-1.310) *p = 0.937*	1.041 (0.604–1.794) *p = 0.885*	0.923 (0.664–1.283) *p = 0.635*	**1.963** **(1.418–2.718)** ***p < 0.001***
Senior	-	1.778 (0.700–4.515) *p = 0.220*	1.316 (0.304–5.706) *p = 0.713*	-	1.095 (0.321–3.740) *p = 0.885*	**3.518** **(1.515–8.166)** ***p = 0.006***	-	1.366 (0.315–5.922) *p = 0.676*	1.351 (0.179–10.174) *p = 0.536*
Design									
RCT	**1.437** **(1.207–1.711)** ***p < 0.001***	**1.253** **(1.165–** **1.349)** ***p < 0.001***	**1.407** **(1.273–1.556)** ***p < 0.001***	**1.272** **(1.128–1.436)** ***p < 0.001***	**1.248** **(1.152–1.351)** ***p < 0.001***	**1.195** **(1.095–1.304)** ***p < 0.001***	**1.408** **(1.180–1.681)** ***p < 0.001***	**1.289** **(1.161–1.431)** ***p < 0.001***	**1.420** **(1.235–1.633)** ***p < 0.001***

*Cells with statistical significance (*P* < 0.05) were highlighted by bold face. *RCT* randomized controlled trial

Phase 0 trials were less prevalent in all nine countries than those in the USA. There was a larger number of phase 1 trials in India (OR 1.517*, p* < 0.001) than in the USA, while phase 3 trials were more prevalent in all nine representative countries compared to the USA, particularly in Poland (OR 3.557, *p* < 0.001) and South Africa (OR 3.349, *p* < 0.001). A larger number of phase 4 trials was conducted in Brazil (OR 2.868, *p* < 0.001), China (OR 2.142, *p* < 0.001), and Korea (OR 1.783, *p* < 0.001) than in the USA.

In addition, compared to the USA, industry sponsorships were significantly higher in all countries except for Brazil (OR 0.592, *p* < 0.001) and China (OR 0.795, *p* < 0.001), while “others” sponsorship type was significantly higher in Brazil (OR 1.534, *p* < 0.001) and China (OR 1.322, *p* < 0.001).

Moreover, the proportion of “adult” age group trials was particularly high in India (OR 2.695, *p* < 0.001). In contrast, a larger number of child age group trials was performed in Poland (OR 1.852, *p* < 0.001), Israel (OR 1.546, *p* = 0.005), and South Africa (OR 1.963, *p* < 0.001), compared to the USA, whereas there were more “senior” age group trials in China (OR 3.518, *p* = 0.006). While nonrandomized controlled trial design was the prevalent form of the study design in the USA, the randomized controlled trial design, i.e., the recommended study design for phase 3 studies, was more dominant in nine representative countries (Table 2).

## Discussion

We analyzed the characteristics of drug intervention clinical trials in 2011–2013 from the top 30 countries obtained at the ClinicalTrials.gov website. The findings from our study indicated that the traditional clinical trial sites of North America and Western Europe were still the locations of the largest number of trials despite the growing tendency of globalization. A consistent increase in the number of clinical trials was observed in the emerging regions of Asia, Eastern Europe, and South America. Among them, Korea, China, and Japan are the leading countries in Asia followed by Russia. Poland, and the Czech Republic are the leading countries in Eastern Europe, while Australia, South Africa, and Israel are the leading countries in the regions of Pacifica, Africa, and Middle East, respectively. The overall number of clinical trials per year decreased from 2011 to 2013, reflecting the global economic marked down-turn from 2011 to early 2013 [24].

There are many factors influencing the selection of clinical trial sites which benefit drug development by pharmaceutical companies, potentially contributing to the globalization of clinical trials. The factors associated with this shift include the ability to reduce operational costs while recruiting a large number of patients; the establishment of contract research organizations focused on global clinical trials; the rapid growth of the market size, research capacity, and regulatory authority in emerging regions; regulatory barriers; widespread adoption of the Good Clinical Practice guidelines by the International Conference on Harmonization of Technical Requirements for Registration of Pharmaceuticals for Human Use; and strong intellectual property protection [2, 22, 25–32]. Published academic literature described that one of the main reasons for sponsor companies to choose developing countries as their new trial locations was cost savings, thus leading them to primarily conduct their phase 2 or 3 trials in places such as China, India, and South America [22]. This theory is corroborated by findings from our study that phase 3 trials are more prevalent in nine representative countries of their respective continental regions than in the USA; on the contrary, there was a smaller number of phase 1 trials in all nine countries than in the USA except for India (OR 1.517 *p* < 0.001). Because of high risk-benefit balance in pharmacokinetic and pharmacodynamic safety trials of phase I trials, it is difficult to recruit healthy volunteers and has high dropout rates [33]. Therefore, a very low operation cost and a large number of volunteering healthy people might help to perform phase 1 studies in India, and this fact can explain the high adult group in India (OR 2.695, *p* < 0.001), as pharmacokinetic studies usually require an 18–55 year-old adult group excluding seniors [14]. In fact, clinical trial business in India amounted to approximately US$1 billion in 2010—an increase from US$200 million in 2009—making India one of the world’s most preferred locations for clinical trials [34].

Another important contributing factor towards globalization is the availability of large, untested research populations who readily volunteer their involvement in clinical trials, thus facilitating and accelerating the recruitment process [35–37] and consequently offering a promising drug market [38]. As observed in our study, there are more phase 4 trials in Brazil and China than in the USA, indicating that there is a targeting of countries with expanding markets associated with larger populations; China has the largest population, while Brazil ranks the 5th in population size. Outside of the USA there are still very few phase 0 clinical trials, a recent designation for exploratory, first-in-human trials by the US FDA [39] which usually require small number of subjects.

Regarding age groups, a larger number of child age group trials was performed in Poland, Israel, and South Africa compared to the USA, whereas there were many more “senior” age group trials in China. Our findings on the studies involving vulnerable populations, like children or senior groups, could reflect the potentially worrying ethical concerns in low- or lower-middle income countries. The US FDA [40] and the European Medicine Agency [41] have regulations on clinical trials involving pediatric populations, Japan is currently enacting its regulation, and the WHO [39] provides pediatric clinical trial guideline on its home page to improve accessibility. The sponsoring pharmaceutical industry should keep up with these guidelines and regulations, and shared effort with the emerging countries is needed to protect the vulnerable population.

One of the strengths of our study is the analytical approach to creating a statistical model to predict regional distribution of the clinical trials. A study by Kerney (2007) [18] introduced the term “Country Attractiveness Index” for clinical trials generated from patient pool, cost efficiency, relevant expertise, regulatory conditions, infrastructure, and environment. However, our study provided a predictive model showing that the distribution of clinical trials was explained by EFI, HEC, HCI, and IPRI, with a satisfactory level of model fitting. In other words, the factors embracing health care infrastructure (i.e., HEC), free trade, low bureaucratic burden (i.e., EFI), provision of high education (i.e., HCI), and intellectual property rights (i.e., IPRI) were sufficiently able to explain the variance of clinical trial distribution. Although the academic literature indicated that the variability of the cost index of clinical trials in each country could partially explain the location of the clinical trials [40], the information was not available in our study. Therefore, further studies are needed to explain other determinants for selection of the clinical trial distribution.

A number of limitations should be considered in interpreting the findings from our study. First, the selection of the top 30 countries by the largest number of clinical trials performed was made only with available trial elements from the data source, which would limit the level of comprehensive understanding for each country. Second, our study included drug intervention trials. Therefore, interpretations of the findings should be limited to drug trials but not for the trials on radiotherapy, procedure, or devices. The research including nonpharmacological trials could be a good comparison study in the future. In addition, the focus of our study was on the global contribution of each country in generating the sheer volume of clinical trials. Therefore, the population-adjusted capacity of each country in generating clinical trial was not explored in this study, generalization and interpretation of our findings should be made with caution in light of these limitations.

## Conclusions

In conclusion, HEC, EFI, HCI, and IPRI were able to explain the geographical distribution of clinical trials. Of note, the comparative characteristics of design elements of clinical trials with those of the USA were quite different and reflected the factors of economic, population, and regulatory issues.

## Additional files

Additional file 1: Definitions of the explanatory variables in regression analysis. (DOC 23 kb)

Additional file 2: The geographical distribution and characteristics of drug interventional clinical trial in the top 30 countries worldwide, 2011–2013. (DOCX 29 kb)

Additional file 3: Number values of associated factors (explanatory variables) considered in regression analysis on clinical trial site distribution. (DOCX 16 kb)

## Abbreviations

EFI: Economic Freedom Index
FDA: Food and Drug Administration
GDP: Gross Domestic Product
HCI: Human Capital Index
HEC: Health Care Expenditure per Capita
IPRI: Intellectual Property Rights Index
PIM: Population In Million
TRDS: Trial Registry Data Set.
WHO: World Health Organization
