# Supplementary material for: Current globalization of drug interventional clinical trials: characteristics and associated factors, 2011–2013
Source: Trials. 2017 Jun 21;18:288. doi: 10.1186/s13063-017-2025-1 (PMC5480138; doi:10.1186/s13063-017-2025-1)
Supplement: Supplementary file 1 — Definitions of the explanatory variables in regression analysis. (DOC 23 kb) [file 13063_2017_2025_MOESM1_ESM.doc]

**Additional file 1. Definitions of the explanatory variables in regression analysis**

1. Population in millions (PIM): The data of population in millions for each country was obtained from the ‘2014 World Population Data Sheet’ and the website indicated that the source were from recent demographic surveys such as the Demographic and Health Surveys, Reproductive Health Surveys, special studies, and direct communication with demographers and statistical bureaus in the United States and abroad^1^
2. Gross Domestic Product (GDP): The data was retrieved from the website of KNOEMA. Domestic product covers different indicators of national accounts with a focus on Gross Domestic Product (GDP). GDP is the standard measure of the value of final goods and services produced by a country during a period minus the value of imports. While GDP is the single most important indicator to capture these economic activities, it is not considered as a good measure of societies' well-being and only a limited measure of people's material living standards^2^.
3. Health care expenditure per capita (HEC): Total health expenditure is the sum of public and private health expenditures as a ratio of total population. It covers the provision of health services (preventive and curative), family planning activities, nutrition activities, and emergency aid designated for health but does not include provision of water and sanitation^3^. Data are in U.S. dollars.
4. Economic freedom Index (EFI) : Economic freedom was measured based on 10 quantitative and qualitative factors, grouped into four broad categories, or pillars, of economic freedom: Rule of Law (property rights, freedom from corruption); Limited Government (fiscal freedom, government spending); Regulatory Efficiency (business freedom, labor freedom, monetary freedom); and Open Markets (trade freedom, investment freedom, financial freedom). Each of the ten economic freedoms within these categories is graded on a scale of 0 to 100. A country’s overall score is derived by averaging these ten economic freedoms, with equal weight being given to each^4^.
5. Human Capital Index (HCI): This index is a measure for capturing and tracking the state of human capital development around the world. The index has four pillars: three core determinants of human capital (Education pillar, Health and Wellness pillar, Workforce and Employment pillar) plus those factors that allow these three core determinants to translate into greater returns(Enabling Environment pillar)^5^
6. Intellectual Property Rights Index (IPRI): World Intellectual Property Indicators is an annual report providing a wide range of indicators covering the areas of intellectual property. It has four categories of Patent, Trademarks and Plant varieties and the indicators used in our study include three categories except for Plant varieties.^6^

1. 2014 World Population Data Sheet. 2014. at <http://www.prb.org/pdf14/2014-world-population-data-sheet_eng.pdf>.)

2. Domestic Product. (Accessed Feb. 22, 2015, at <http://data.oecd.org/gdp/gross-domestic-product-gdp.htm>.)

3. Health expenditure per capita. 2015. at <http://data.worldbank.org/indicator/SH.XPD.PCAP>.)

4. 2015 Index of Economic Freedom. 2015. at <http://www.heritage.org/index/ranking>.)

5. The Human Capital Report. 2013. at <http://www3.weforum.org/docs/WEF_HumanCapitalReport_2013.pdf>.)

6. World Intellectual Property Indicators, 2014. WIPO, 2014. (Accessed Feb.22, 2015, at <http://www.wipo.int/edocs/pubdocs/en/wipo_pub_941_2014.pdf>.)
