# Supplementary material for: Current globalization of drug interventional clinical trials: characteristics and associated factors, 2011–2013
Source: Trials. 2017 Jun 21;18:288. doi: 10.1186/s13063-017-2025-1 (PMC5480138; doi:10.1186/s13063-017-2025-1)
Supplement: Supplementary file 2 — The geographical distribution and characteristics of drug interventional clinical trial in the top 30 countries worldwide, 2011–2013. (DOCX 29 kb) [file 13063_2017_2025_MOESM2_ESM.docx]

**Additional file 2. The geographical distribution and characteristics of drug interventional clinical trial in the top thirty countries worldwide, 2011-2013**

| **Country** | US (10473) | DE (2079) | FR (1863) | CA  (1859) | GB  (1787) | KR (1688) | ES (1540) | CN (1401) | IT (1333) | BE  (1036) | JP (1016) | PL  (936) | AU (902) | RU (808) | IL  (660) | CZ  (657) | TW (640) | DK (619) | SE  (582) | AT  (571) | CH (463) | ZA (456) | GR (315) | FI (314) | BR (285) | IN (280) | SG  (277) | NL (264) | HK (198) | NO (91) | Total  (35393) |
| --- | --- | --- | --- | --- | --- | --- | --- | --- | --- | --- | --- | --- | --- | --- | --- | --- | --- | --- | --- | --- | --- | --- | --- | --- | --- | --- | --- | --- | --- | --- | --- |
| **Phase** |  |  |  |  |  |  |  |  |  |  |  |  |  |  |  |  |  |  |  |  |  |  |  |  |  |  |  |  |  |  |  |
| 0 | 145 | 0 | 1 | 5 | 5 | 3 | 1 | 11 | 3 | 3 | 0 | 0 | 1 | 0 | 5 | 0 | 3 | 3 | 2 | 0 | 3 | 0 | 0 | 0 | 1 | 0 | 0 | 1 | 0 | 0 | 196 |
| 1 | 2565 | 371 | 212 | 195 | 415 | 271 | 121 | 130 | 59 | 160 | 196 | 29 | 125 | 32 | 36 | 22 | 35 | 52 | 34 | 30 | 67 | 32 | 8 | 22 | 23 | 104 | 80 | 68 | 11 | 5 | 5510 |
| 2 | 3018 | 612 | 566 | 515 | 464 | 348 | 446 | 329 | 391 | 274 | 202 | 278 | 231 | 182 | 130 | 181 | 146 | 139 | 151 | 154 | 105 | 106 | 66 | 62 | 33 | 38 | 58 | 48 | 44 | 25 | 9342 |
| 3 | 1639 | 760 | 723 | 728 | 611 | 518 | 669 | 350 | 593 | 451 | 477 | 524 | 419 | 501 | 289 | 392 | 289 | 245 | 268 | 289 | 178 | 239 | 171 | 162 | 87 | 49 | 100 | 52 | 105 | 21 | 11899 |
| 4 | 1089 | 176 | 145 | 199 | 156 | 313 | 180 | 312 | 185 | 77 | 54 | 80 | 68 | 62 | 73 | 42 | 102 | 93 | 58 | 61 | 61 | 49 | 45 | 47 | 85 | 32 | 20 | 42 | 18 | 27 | 3951 |
| 1 and 2 | 740 | 82 | 104 | 67 | 81 | 47 | 68 | 57 | 48 | 42 | 31 | 10 | 33 | 16 | 29 | 9 | 16 | 16 | 24 | 16 | 26 | 8 | 4 | 4 | 5 | 8 | 7 | 15 | 7 | 7 | 1627 |
| 2 and 3 | 181 | 40 | 40 | 28 | 25 | 24 | 37 | 62 | 26 | 14 | 31 | 9 | 16 | 12 | 14 | 7 | 13 | 15 | 10 | 7 | 5 | 16 | 10 | 9 | 15 | 8 | 4 | 10 | 3 | 1 | 692 |
| N/A | 1096 | 38 | 72 | 122 | 30 | 164 | 18 | 150 | 28 | 15 | 25 | 6 | 9 | 3 | 84 | 4 | 36 | 56 | 35 | 14 | 18 | 6 | 11 | 8 | 36 | 41 | 8 | 28 | 10 | 5 | 2176 |
| **Recruitment status** |  |  |  |  |  |  |  |  |  |  |  |  |  |  |  |  |  |  |  |  |  |  |  |  |  |  |  |  |  |  |  |
| completed | 3238 | 806 | 451 | 495 | 692 | 602 | 358 | 383 | 287 | 356 | 468 | 342 | 221 | 232 | 118 | 224 | 171 | 209 | 190 | 188 | 112 | 157 | 72 | 110 | 95 | 181 | 89 | 113 | 33 | 34 | 11027 |
| terminated | 533 | 92 | 66 | 75 | 78 | 37 | 64 | 16 | 51 | 44 | 22 | 54 | 43 | 30 | 25 | 34 | 29 | 25 | 19 | 29 | 23 | 20 | 15 | 7 | 5 | 7 | 8 | 14 | 13 | 3 | 1481 |
| recruiting | 4580 | 758 | 904 | 793 | 597 | 781 | 716 | 738 | 651 | 357 | 302 | 279 | 401 | 309 | 279 | 203 | 315 | 250 | 239 | 204 | 224 | 166 | 149 | 133 | 89 | 62 | 136 | 93 | 98 | 32 | 14838 |
| withdrawn | 161 | 14 | 8 | 19 | 16 | 9 | 12 | 9 | 5 | 8 | 1 | 7 | 7 | 5 | 7 | 5 | 2 | 5 | 2 | 3 | 3 | 6 | 0 | 0 | 5 | 3 | 3 | 3 | 1 | 1 | 330 |
| Active, not recruiting | 1464 | 365 | 376 | 370 | 349 | 190 | 337 | 156 | 290 | 245 | 206 | 234 | 201 | 217 | 122 | 181 | 100 | 105 | 114 | 135 | 82 | 95 | 70 | 54 | 21 | 13 | 38 | 19 | 45 | 10 | 6204 |
| Enrolling by invitation | 116 | 13 | 19 | 25 | 19 | 18 | 17 | 19 | 10 | 12 | 9 | 12 | 15 | 9 | 13 | 8 | 7 | 9 | 8 | 6 | 6 | 9 | 1 | 4 | 4 | 4 | 0 | 2 | 4 | 1 | 399 |
| Not yet recruiting | 304 | 20 | 34 | 70 | 30 | 50 | 30 | 76 | 32 | 12 | 7 | 7 | 11 | 5 | 95 | 2 | 16 | 13 | 7 | 5 | 10 | 2 | 4 | 5 | 64 | 9 | 1 | 18 | 3 | 10 | 952 |
| Suspended | 77 | 7 | 5 | 12 | 6 | 1 | 6 | 4 | 3 | 2 | 1 | 1 | 3 | 1 | 1 | 0 | 0 | 3 | 1 | 1 | 3 | 1 | 0 | 1 | 2 | 1 | 2 | 2 | 1 | 0 | 148 |
| others | 0 | 4 | 0 | 0 | 0 | 0 | 0 | 0 | 4 | 0 | 0 | 0 | 0 | 0 |  | 0 | 0 | 0 | 2 | 0 | 0 | 0 | 4 | 0 | 0 | 0 | 0 | 0 | 0 | 0 | 14 |
| **Sponsor type** |  |  |  |  |  |  |  |  |  |  |  |  |  |  |  |  |  |  |  |  |  |  |  |  |  |  |  |  |  |  |  |
| Industry | 5028 | 1657 | 1262 | 1225 | 1433 | 1006 | 1215 | 535 | 956 | 873 | 924 | 888 | 785 | 765 | 439 | 636 | 470 | 367 | 426 | 438 | 290 | 400 | 240 | 230 | 81 | 169 | 232 | 120 | 170 | 25 | 23285 |
| NIH, U.S federation | 558 | 2 | 4 | 41 | 3 | 2 | 1 | 2 | 0 | 0 | 1 | 0 | 8 | 0 | 1 | 0 | 1 | 0 | 0 | 0 | 2 | 7 | 0 | 0 | 0 | 1 | 4 | 1 | 0 | 0 | 639 |
| others | 4887 | 420 | 597 | 593 | 351 | 680 | 324 | 864 | 377 | 163 | 91 | 48 | 109 | 43 | 220 | 21 | 169 | 252 | 156 | 133 | 171 | 49 | 75 | 84 | 204 | 110 | 41 | 143 | 28 | 66 | 11469 |
| **Age** |  |  |  |  |  |  |  |  |  |  |  |  |  |  |  |  |  |  |  |  |  |  |  |  |  |  |  |  |  |  |  |
| Adult/senior | 6698 | 1469 | 1387 | 1305 | 1189 | 1139 | 1181 | 871 | 1008 | 719 | 744 | 698 | 673 | 611 | 451 | 528 | 504 | 464 | 412 | 430 | 336 | 311 | 249 | 207 | 133 | 66 | 189 | 146 | 170 | 63 | 24351 |
| Child/adult/senior | 511 | 85 | 98 | 99 | 93 | 73 | 70 | 74 | 70 | 52 | 73 | 48 | 48 | 45 | 36 | 29 | 27 | 25 | 45 | 24 | 16 | 34 | 15 | 12 | 22 | 23 | 10 | 4 | 12 | 5 | 1778 |
| Child/adult | 468 | 48 | 61 | 92 | 53 | 38 | 43 | 71 | 47 | 32 | 19 | 31 | 30 | 20 | 40 | 14 | 13 | 19 | 16 | 20 | 10 | 9 | 9 | 12 | 13 | 13 | 8 | 6 | 1 | 3 | 1259 |
| adult | 2276 | 371 | 207 | 257 | 365 | 355 | 145 | 309 | 108 | 171 | 136 | 77 | 109 | 79 | 84 | 54 | 70 | 81 | 77 | 71 | 81 | 58 | 27 | 57 | 104 | 164 | 62 | 89 | 9 | 18 | 6071 |
| child | 503 | 100 | 95 | 103 | 83 | 80 | 94 | 68 | 94 | 59 | 44 | 80 | 40 | 53 | 49 | 32 | 26 | 29 | 29 | 24 | 18 | 43 | 15 | 26 | 13 | 14 | 8 | 19 | 6 | 1 | 1848 |
| senior | 17 | 6 | 15 | 3 | 4 | 3 | 7 | 8 | 6 | 3 | 0 | 2 | 2 | 0 | 0 | 0 | 0 | 1 | 3 | 2 | 2 | 1 | 0 | 0 | 0 | 0 | 0 | 0 | 0 | 1 | 86 |

***Abbreviation of country name by ISO**: US(United States),DE(Germany),FR(France),CA(Canada),GB(UnitedKingdom),KR(Korea),ES(Spain),CN(China),IT(Italy),BE(Belgium),JP(Japan), PL(Poland),AU(Australia),RU(Russia),IL(Israel),CZ(Czech Republic),TW(Taiwan),DK(Denmark),SE(Sweden),AT(Austria),CH(Switzerland),ZA(South Africa), GR(Greece), FI(Finland), BR(Brazil), IN(India), SG(Singapore), NL(Netherlands),HK(Hong Kong), NO(Norway)
