# Supplementary material for: Current globalization of drug interventional clinical trials: characteristics and associated factors, 2011–2013
Source: Trials. 2017 Jun 21;18:288. doi: 10.1186/s13063-017-2025-1 (PMC5480138; doi:10.1186/s13063-017-2025-1)
Supplement: Supplementary file 3 — Number values of associated factors (explanatory variables) considered in regression analysis on clinical trial site distribution. (DOCX 16 kb) [file 13063_2017_2025_MOESM3_ESM.docx]

Additional file 3. Number values of associated factors (explanatory variables) considered in regression analysis on clinical trial globalization

|  | Variables | | | | | | |
| --- | --- | --- | --- | --- | --- | --- | --- |
| Country | NCT | PIM | GDP | EFI | HEC | HCI  (rank) | IPRI |
| US | 10473 | 317.7 | 17,416 | 76.2 | 8,895 | 16 | 10 |
| DE | 2079 | 80.9 | 3,820 | 73.8 | 4,683 | 6 | 11 |
| FR | 1863 | 64.1 | 2,902 | 62.5 | 4,690 | 21 | 18 |
| CA | 1859 | 35.5 | 1,794 | 79.1 | 5,741 | 10 | 52 |
| GB | 1787 | 64.5 | 2,848 | 75.8 | 3,647 | 8 | 26 |
| KR | 1688 | 50.4 | 1,450 | 71.5 | 1,703 | 23 | 17 |
| ES | 1540 | 46.5 | 1,401 | 67.6 | 2,808 | 29 | 46 |
| CN | 1401 | 1,364.1 | 10.355 | 52.7 | 322 | 43 | 3 |
| IT | 1333 | 61.3 | 2,129 | 61.7 | 3,032 | 37 | 27 |
| BE | 1036 | 11.2 | 528 | 68.8 | 4,711 | 11 | 85 |
| JP | 1016 | 127.1 | 4,770 | 73.3 | 4,752 | 15 | 15 |
| PL | 936 | 38.5 | 552 | 68.6 | 854 | 49 | 65 |
| AU | 902 | 23.5 | 1,483 | 81.4 | 6,140 | 19 | 51 |
| RU | 808 | 143.7 | 2,057 | 52.1 | 887 | 51 | 36 |
| IL | 660 | 8.2 | 305 | 70.5 | 2,289 | 25 | 121 |
| CZ | 657 | 10.5 | 200 | 72.5 | 1,432 | 33 | 91 |
| TW | 640 | 23.4 | 506 | 75.1 | N/A | N/A | N/A |
| DK | 619 | 5.6 | 347 | 76.3 | 6,304 | 9 | 78 |
| SE | 582 | 9.7 | 559 | 72.7 | 5,319 | 5 | 54 |
| AT | 571 | 8.5 | 436 | 71.2 | 5,407 | 13 | 49 |
| CH | 463 | 8.2 | 679 | 80.5 | 8,980 | 1 | 28 |
| ZA | 456 | 53.7 | 341 | 62.6 | 645 | 86 | 113 |
| GR | 315 | 11.0 | 246 | 54.0 | 2,044 | 55 | 153 |
| FI | 314 | 5.5 | 276 | 73.4 | 4,232 | 2 | 85 |
| BR | 285 | 202.8 | 2,244 | 56.6 | 1,056 | 57 | 56 |
| IN | 280 | 1,296.2 | 2,048 | 54.6 | 61 | 78 | 38 |
| SG | 277 | 5.5 | 307 | 89.4 | 2,426 | 3 | 101 |
| NL | 264 | 16.9 | 880 | 73.7 | 5,737 | 4 | 45 |
| HK | 198 | 7.2 | 293 | 89.6 | NA | N/A | 88 |
| NO | 91 | 5.1 | 512 | 71.8 | 9,055 | 7 | 124 |

**#Abbreviation of country name by ISO**: US(USA),DE(Germany),FR(France),CA(Canada), GB(UnitedKingdom),KR(Korea),ES(Spain),CN(China),IT(Italy),BE(Belgium),JP(Japan),PL(Poland),AU(Australia),RU(Russia),IL(Israel),CZ(Czech),TW(Taiwan),DK(Denmark),SE(Sweden),AT(Austria),CH(Switzerland),ZA(South Africa), GR(Greece), FI(Finland), BR(Brazil), IN(India), SG(Singapore), NL(Netherlands),HK(Hong Kong), NO(Norway)

**##Variable**: NCT (number of clinical trials), PIM (population in millions), GDP (gross domestic product), EFI (economic freedom index), HEC (healthcare expenditure per capita), HCI (human capital index), IPRI (intellectual property rights index)
